# Myosins VIII and XI Play Distinct Roles in Reproduction and Transport of *Tobacco Mosaic Virus*

**DOI:** 10.1371/journal.ppat.1004448

**Published:** 2014-10-16

**Authors:** Khalid Amari, Martin Di Donato, Valerian V. Dolja, Manfred Heinlein

**Affiliations:** 1 Zürich-Basel Plant Science Center, Botany, Department of Environmental Sciences, University of Basel, Basel, Switzerland; 2 Department of Botany and Plant Pathology and Center for Genome Research and Biocomputing, Oregon State University, Corvallis, Oregon, United States of America; 3 Institut de Biologie Moléculaire des Plantes du CNRS, Université de Strasbourg, Strasbourg, France; University of Kentucky, United States of America

## Abstract

Viruses are obligatory parasites that depend on host cellular factors for their replication as well as for their local and systemic movement to establish infection. Although myosin motors are thought to contribute to plant virus infection, their exact roles in the specific infection steps have not been addressed. Here we investigated the replication, cell-to-cell and systemic spread of *Tobacco mosaic virus* (TMV) using dominant negative inhibition of myosin activity. We found that interference with the functions of three class VIII myosins and two class XI myosins significantly reduced the local and long-distance transport of the virus. We further determined that the inactivation of myosins XI-2 and XI-K affected the structure and dynamic behavior of the ER leading to aggregation of the viral movement protein (MP) and to a delay in the MP accumulation in plasmodesmata (PD). The inactivation of myosin XI-2 but not of myosin XI-K affected the localization pattern of the 126k replicase subunit and the level of TMV accumulation. The inhibition of myosins VIII-1, VIII-2 and VIII-B abolished MP localization to PD and caused its retention at the plasma membrane. These results suggest that class XI myosins contribute to the viral propagation and intracellular trafficking, whereas myosins VIII are specifically required for the MP targeting to and virus movement through the PD. Thus, TMV appears to recruit distinct myosins for different steps in the cell-to-cell spread of the infection.

## Author Summary

Viruses are parasites that require the host cell machinery for their propagation within and between cells. Myosins are molecular motors involved in the trafficking of cargos along actin filaments. Plant viruses have evolved to borrow this transport mechanism to aid their infection and spread within the plant. However, little is known about which of the many plant myosins are essential and at which specific steps they act to support virus infection. Here we investigated the role of different *N. benthamiana* myosins during the infection by *Tobacco mosaic virus* (TMV). Our results show that class XI myosins play specific roles in the reproduction and intracellular movement of TMV in association with the dynamic endoplasmic reticulum network, whereas class VIII myosins support the specific targeting of the viral movement protein to plasmodesmata and thus the cell-to-cell movement of the virus. Together these results indicate that TMV interacts with distinct myosins during specific infection steps.

## Introduction

Viruses are obligate intracellular parasites that depend on host cell functions for replication and movement. Upon infection of a plant cell, viruses replicate and spread to the adjacent cells (cell-to-cell movement) to reach the phloem and infect distal parts of the plant (systemic movement). Cell-to-cell movement occurs through specialized channels in the cell wall called plasmodesmata (PD). PD provide symplastic continuity of the cytoplasm, endoplasmic reticulum (ER) and the plasma membrane (PM) between cells and regulate the intercellular transport of macromolecules by dilation or closure, i.e. through modification of their size exclusion limit (SEL) [1], [2]. Plant viruses have evolved specialized movement proteins (MP) that target PD and facilitate the spread through these channels by different mechanisms [3]. The MP of *Tobacco mosaic virus* (TMV) exemplifies a widespread mechanism in which the MP acts as a chaperone for the viral RNA and increases the SEL of PD. Another important mechanism involves reorganization of the PD structure and is exemplified by *Grapevine fanleaf virus* (GFLV). The MP of this virus assembles into tubules within the PD cavity and thus forms a dedicated transport structure for the passage of assembled virions [4], [5]. In contrast to GFLV, which moves between cells as virion, TMV achieves cell-to-cell movement as a viral ribonucleoprotein complex (vRNP) that contains the viral RNA, MP and possibly also the viral replicase [6]–[8]. The mechanism by which the MP alone or in association with the vRNP is targeted to PD is extensively studied. The MP is peripherally associated with the ER [9] and might target PD with support of actomyosin system and microtubules [2], [10]. The role of actin filaments and actomyosin-mediated transport of TMV to and through PD has been investigated mostly relying on the pharmacological disruption of actin filaments under different experimental conditions, which produced controversial results likely due to pleotropic effects of the inhibitors [11]–[14].

Phylogenetic analysis revealed that all plants encode two distantly related classes of unconventional myosins, VIII and XI, with reference plant Arabidopsis possessing four myosins VIII and 13 myosins XI [15]. The functional roles of myosins VIII in cell growth were established only for moss [16], whereas no genetic evidence is available so far for flowering plants. However, it was suggested that myosins VIII are associated with PD, PM and the ER [17]–[19] and participate in PD function, cell plate formation, and endocytotic processes [19]–[21].

In contrast, functions of the myosins XI are relatively well investigated using dominant negative inhibition and gene knockout approaches. It was found that several of these myosins provide overlapping contributions to both the polar and diffuse cell growth, as well as to plant growth and development [22]–[27]. Myosins XI were also implicated in the saltatory trafficking of Golgi stacks, mitochondria and peroxisomes [23], [24], [28], [29], in the localized flow of the ER network [30], and in nuclear repositioning [31]. It was demonstrated that simultaneous inactivation of the four highly expressed myosins XI results in virtual arrest of organelle trafficking traditionally defined as cytoplasmic streaming [26]. Recently, a novel vesicular transport compartment conserved in all land plants and proposed to be associated with most of the myosin XI functions in cell and plant growth was identified [32], [33]. A very rapid transport of this compartment along the filamentous actin is dependent on interaction between myosins XI and their cognate MyoB family receptors that are anchored in vesicular membranes.

Several studies indicated that myosins exert important functions in the PD targeting of viruses. First experimental support came from Avisar et al. [34] who showed that inactivation of class VIII myosins impaired the PD localization of the Hsp70 (Heat shock protein, 70 kDa) homolog of *Beet yellows closterovirus*, a virion component required for the viral cell-to-cell movement. Subsequent studies showed that other diverse viruses also require either class VIII or class XI myosins for their movement [5], [35]–[38]. A role of myosin XI-2 in the intercellular movement of TMV was shown by Harries et al. [39], although potential contributions of other myosins remained less clear, primarily due to the functional redundancy of myosins [24]–[26], [29], [40].

To gain deeper mechanistic insight into the contributions of myosin motors to TMV infection, we investigated the roles of class VIII and XI myosins using dominant negative inhibition, which is achieved by overexpression of the myosin tails encompassing dimerization and cargo-binding domains. Although the mechanistic details of dominant-negative inhibition are not entirely understood, the inhibitory effect is likely caused by the retention of the cargo-binding activity and lack of the head domain possessing the motor and actin-binding activities. Thus, in binding cargo but not actin the expressed myosin tails sequester the cargo without contributing to its transport. In addition, by forming dimers with endogenous myosins without contributing a motor domain, the expressed tails may dominantly block the processivity of the associated myosin and, thus, cargo transport along the actin filaments. Dominant negative inhibition generally results in stronger functional effects than inactivation of individual myosins [23], [28], likely because overexpression of cargo-binding domain interferes with functions of several functionally redundant myosin paralogs, whereas gene knockout analysis involves the inactivation of only single myosin genes. Using dominant negative inhibition, we show that the myosins XI-2, XI-K, VIII-1, VIII-2 and VIII-B play distinct functional roles in the intracellular transport of viral proteins and virus accumulation, as well as in the cell-to-cell movement of the virus through PD.

## Results

### Both class VIII and class XI myosin motors are involved in the spread of TMV infection

**Figure 1:**
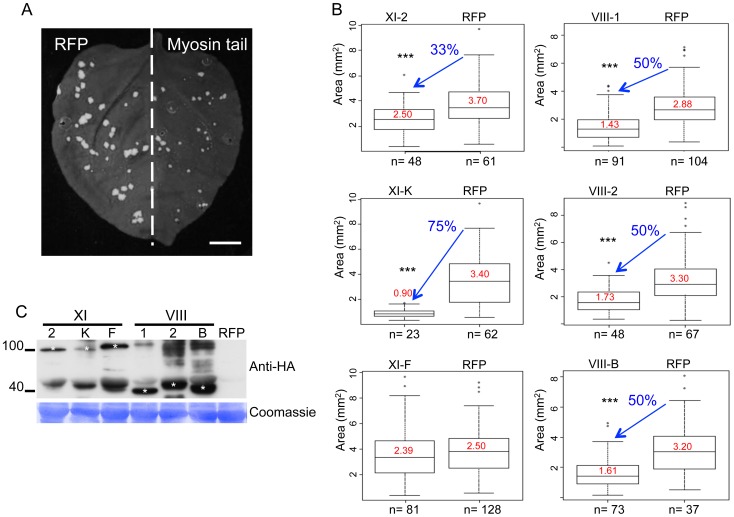
Dominant negative repression of specific myosins inhibits TMV-GFP cell-to-cell spread. A, example of an *N. benthamiana* leaf with TMV-GFP infection sites at 4 dpi in the presence of RFP in one half of the leaf and of inhibitory myosin VIII-1 tails in the other half. B, areas of viral infection sites are reduced in *N. benthamiana* half-leaves transiently expressing myosin VIII-1, VIII-2, VIII-B, XI-2 or XI-K tails as compared to half-leaves expressing RFP. Transient expression of myosin XI-F tails has no effect on the area of TMV-GFP viral infection sites. Boxplots depict the 25^th^ to 75^th^ percentile of measured areas of viral infection sites. Error bars indicate the range of the 5^th^ and 95^th^ percentile of measured areas of viral infection sites. The horizontal bar in each boxplot indicates the median value. Circles above the graphs represent outliers. n represents the number of viral infection sites measured on three half-leaves. Mean area of viral infection sites is given in red. (***) represents significant differences as determined by unbalanced ANOVA (p<0.001). Data are from three independent experiments. C, immunoblot analysis using HA- (top panel) antibodies revealed the expression of the HA-tagged myosin tails. Bands corresponding to class XI (≈100 kDa) and class VIII (≈40 kDa) myosin tails are marked by asterisks. Coomassie blue staining (bottom panel) is shown as loading control. All leaves were inoculated with equal volumes of TMV-GFP in-vitro transcription reaction mix and agro-infiltrated for myosin tail/RFP expression two days later. Analyses were conducted at 4 dpi.

To identify myosin motors involved in TMV movement, *N. benthamiana* plants were inoculated with TMV-GFP, an engineered TMV variant expressing GFP in place of the CP [41]. At two days post inoculation (dpi), the inoculated leaves where agro-infiltrated for expression of a specific *N. benthamiana* myosin tail in one half of the leaf blade and for expression of free RFP as control in the other half (Figure 1A). At 4 dpi, the sizes of fluorescent infection sites were measured and compared between the different treatments. Figure 1B shows that the mean area of TMV-GFP infection sites in the half leaves expressing myosin tails VIII-1, VIII-2, or VIII-B was reduced by ∼50% as compared to the corresponding control half leaves. The sizes of the TMV-GFP infection sites were also reduced upon overexpression of myosin XI-K and XI-2 tails, of which the former had a stronger effect than the latter (Figure 1B). In contrast, overexpression of the myosin XI-F tail did not exert a statistically significant change in the size of virus infection sites. The expression of myosin tails in infected leaves at 4 dpi was validated by immunoblot analysis that clearly showed that the observed effects on TMV spread were dependent on each myosin's identity rather than on differential expression levels (Figure 1C). Using the same assays, similar reductions in the sizes of viral infection sites upon transient expression of myosin tails were observed using TMV-GFP-JL24 [42], a TMV variant that expresses both the GFP and CP.

**Figure 2:**
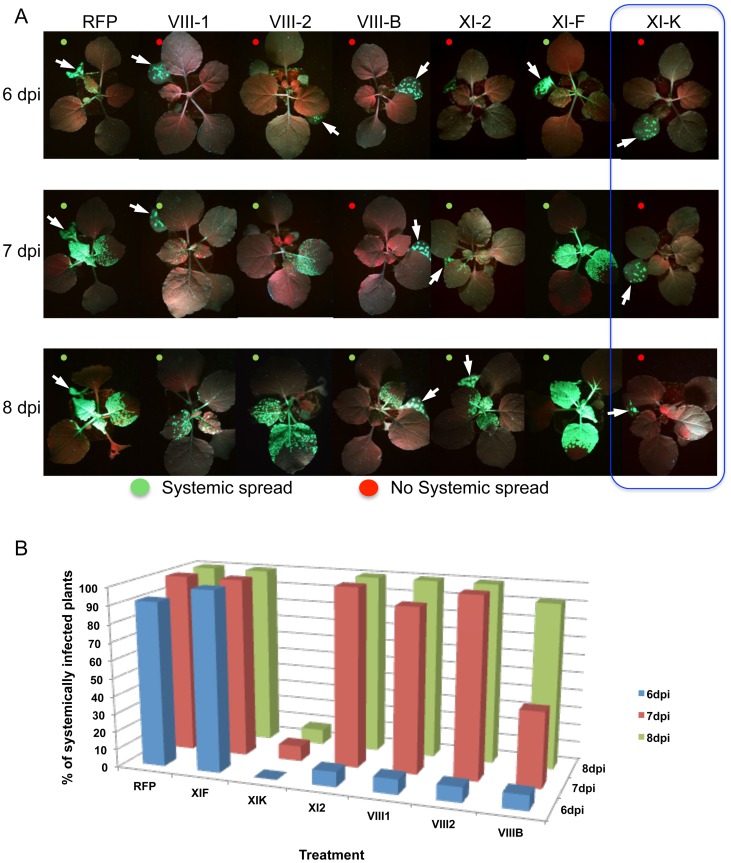
Transient expression of specific myosin tails in the inoculated leaf delays or inhibits the onset of systemic spread of TMV-GFP-JL24. A, the onset of systemic spread is delayed upon transient expression of myosin tails XI-2, VIII-B, VIII-1 and VIII-2 in the inoculated leaf (arrow), whereas expression of XI-F tails has no effect. Overexpression of myosin tails XI-K inhibits the systemic movement of TMV. Images are representative for the results obtained in three independent experiments, each with four plants for each treatment. Single leaves of each plant were inoculated with equal amounts of TMV-GFP-JL24 virions and agroinfiltrated for myosin tail/RFP expression two days later. Plants were scored for systemic infection at 6, 7 and 8 dpi. B, percentage of systemically infected plants at 6, 7 and 8 dpi.

The inhibition of local movement was also accompanied by a delay in the virus entry into the upper, non-inoculated leaves. Whereas TMV-GFP-JL24 infection in plants transiently expressing RFP or myosin XI-F tail in the inoculated leaf appeared in upper leaves at 6 dpi, plants expressing myosin VIII-1, VIII-2 or myosin XI-2 tails showed systemic spread of the virus only at 7 dpi. Plants expressing myosin VIII-B tail in the inoculated leaf showed an even longer delay of 2 days. Strikingly, expression of myosin XI-K tail resulted in abolishment of the systemic spread of TMV-GFP during the entire 8 days-long course of the experiment (Figure 2).

**Figure 3:**
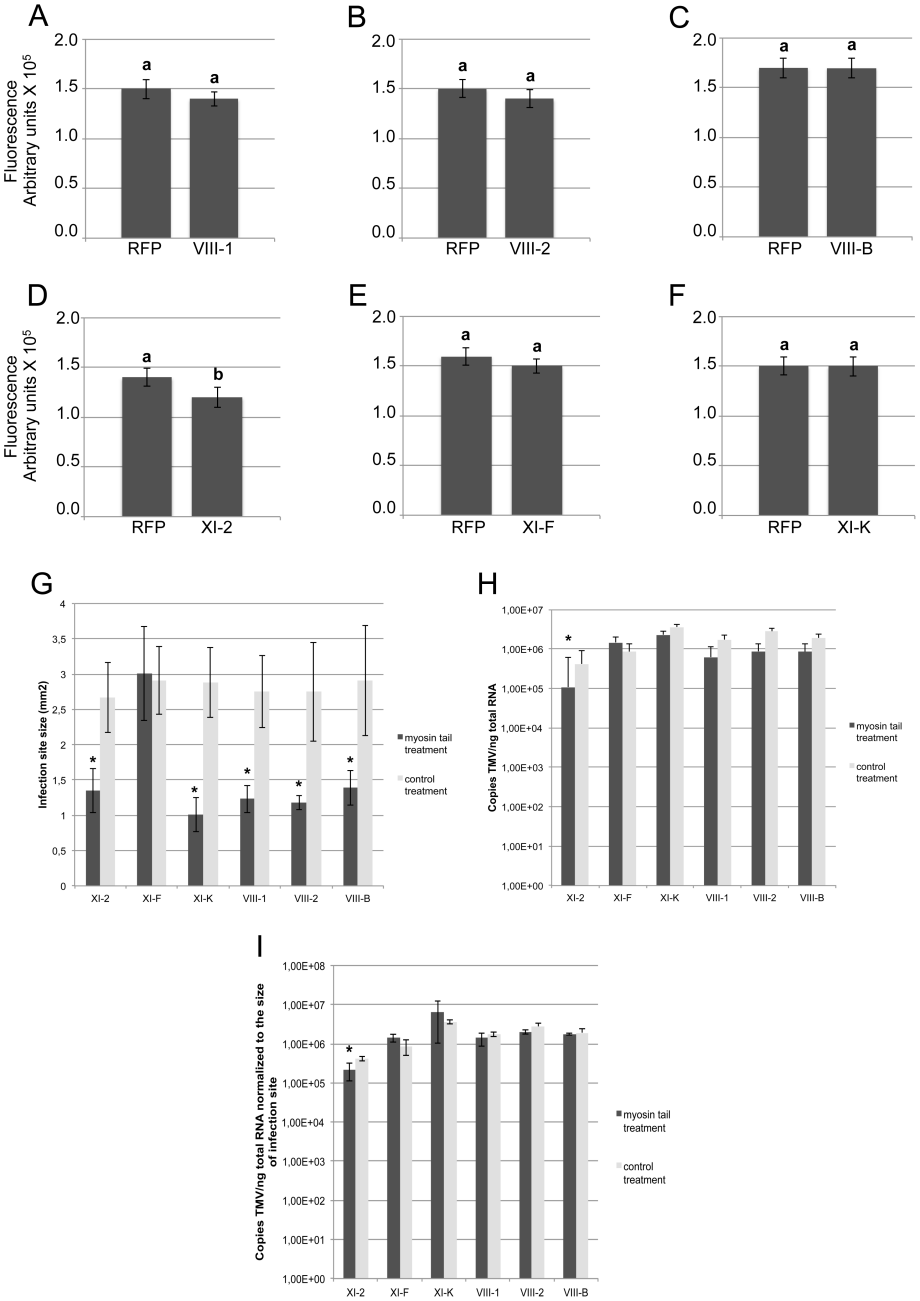
Transient expression of myosin tail XI-2, but not the expression of any of the other tested myosin tails, affects virus replication. A-F, Fluorescence intensity (arbitrary units) of the TMV-GFP infection sites analyzed in Figure 1. Fluorescence intensity was quantified using ImageJ software and normalized to the area of the same infection sites. Except for myosin XI-2 tails (Figure 3D), myosin tail expression had no significant effect on the fluorescence intensity of infection sites (Figure 3A-C, E and F). Different letters (a and b) above the columns indicate statistically significant differences between the different treatments, as determined by *t*-test (P<0.001). G-I, quantification of TMV-GFP RNA upon overexpression of myosin tails. Plants were inoculated with equal amount of TMV-GFP and agro-infiltrated for expression with myosin tails/RFP two days later. Analyses of infection sites were conducted at 4 dpi. At least 38 infection sites from three different leaves taken from three different plants was analysed per treatment. G, size of TMV-GFP infection sites upon expression of myosin tails compared to the control (RFP). With exception of myosin XI-F tails, the expression of all other tested myosin tails significantly reduced the area of the infection sites. H, quantification of TMV-GFP RNA accumulation within individual infection sites upon myosin tail expression. For all treatments, the same amount of leaf material carrying three infection sites was used for total RNA extraction. The statistical analysis is based on three independent experiments (three biological replicates). Overexpression of myosin XI-2 tails reduces the accumulation of TMV-GFP, whereas the expression of all other test myosin tails had no effect. I, copies of TMV-GFP genomes as shown in Figure 3H normalized to the area of the respective infection sites in Figure 3G. Among the tested myosin tails, only myosin XI-2 tails affect viral RNA accumulation. Statistical significance was determined by t-test (* = P<0.05). Bars represent means and standard errors.

The observed effects of the myosin tails on the spread of TMV infection could be attributed either to a defect in virus transport between cells, or to a reduction in virus accumulation. To address the latter possibility, we quantified TMV-GFP fluorescence intensity of the infection sites in the inoculated leaves as a surrogate marker of the virus replication and gene expression levels. To use this marker as a measure for the level of viral accumulation and gene expression per cell, the fluorescence intensity of each site was normalized relative to the size of this site. Figure 3A-F shows that the overexpression of XI-2 myosin tail caused a moderate, but statistically significant decrease of the fluorescence (Figure 3D), whereas the fluorescence intensities of sites expressing tails of myosins XI-K, XI-F, VIII-1; VIII-2 or VIII-B were not significantly different from that in the RFP-expressing control sites. To confirm this apparent effect of myosin XI-2 tail on TMV accumulation, we quantified viral RNA within individual infection sites by quantitative RT-PCR and normalized the levels to the amount of total RNA and to the area of the respective infection site (Figure 3G-I). As shown in Figure 3I, the normalized number of TMV-GFP genome copies is decreased upon expression of myosin XI-2 tails. Thus, among the tested myosins, only myosin XI-2 appears to affect a steady state level of TMV RNA. Together, these results indicate that both class VIII and class XI myosin motors contribute to efficient cell-to-cell movement of TMV. Moreover, myosin XI-2, but not XI-K, XI-F, or any of the three tested class VIII myosins, may contribute to virus movement by exerting a positive effect on virus accumulation.

### Inhibition of myosins XI-2 and XI-K affects the subcellular localization of MP and the structure and dynamic behavior of the ER network

**Figure 4:**
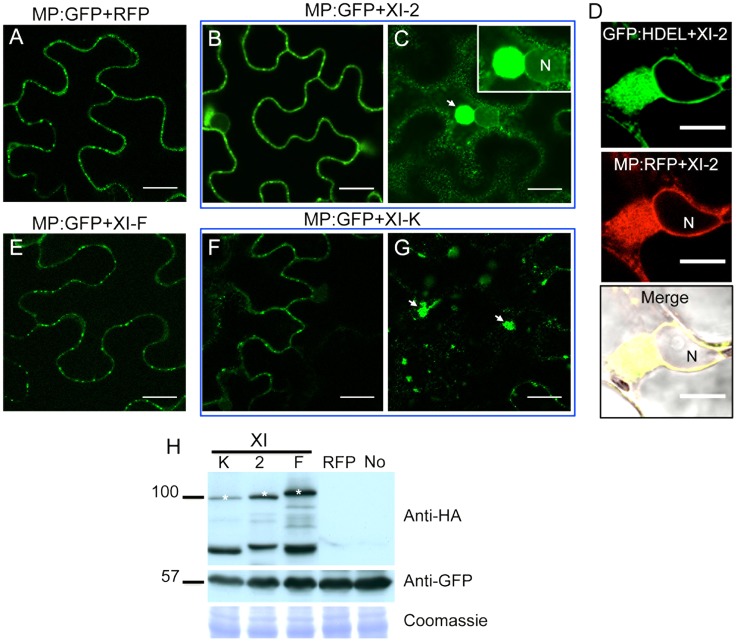
Transient expression of myosin XI-2 and XI-K tails changes the accumulation pattern of the MP. A, co-expression of MP:GFP with RFP. The MP:GFP accumulates in PD. B and C, co-expression of the MP:GFP together with myosin XI-2 tails does not affect significantly the localization of the MP:GFP to PD (B) but causes the accumulation of the MP:GFP in a single big aggregate in the vicinity of the nucleus (C, arrow). D, transient co-expression of myosin XI-2 tails and MP:RFP in *N. benthamiana* transgenic plants expressing GFP:HDEL as ER marker (16c). Accumulation of the MP:RFP in a single aggregate near to the nucleus upon inactivation of myosin XI-2 correlates with the accumulation of the GFP:HDEL indicating that the MP aggregates are ER-derived. E, co-expression of the MP:GFP with myosin XI-F tails. The MP:GFP accumulates in PD similar as in the control with RFP (A). F and G, co-expression of the MP:GFP together with myosin XI-K tails has no significant effect on the localization of the MP:GFP to PD (F) but causes accumulation of the MP:GFP in several aggregates (G, arrow). H, immunoblot analysis using HA-specific (top panel) and GFP-specific (middle panel) antibodies for the detection of myosin and MP:GFP, respectively. Bands corresponding to the class XI (≈100 kDa) myosin tails are marked by asterisks. Coomassie blue staining (bottom panel) is shown as loading control. A-H, proteins were expressed by co-agroinfiltration and observed at 1 dpa. N, Nucleus. No, MP:GFP expressed alone. Scale bars, 20 µm (A-C and E-G) and 10 µm (D).

To further address the mechanisms whereby myosin motors contribute to TMV cell-to-cell movement, we analyzed the effects of myosin tail expression on the subcellular localization of the viral MP using transient expression of MP fused to GFP at the C-terminus (MP:GFP). As expected, no effect on the subcellular distribution of MP:GFP was observed upon co-expression with either RFP or with the myosin XI-F tails (Figure 4A and E). Although co-expression of MP:GFP with the tails of myosins XI-2 or XI-K had no obvious effect on the PD targeting of MP:GFP (Figure 4B and F), it caused the accumulation of the MP:GFP at aberrant intracellular sites. In the case of XI-2 tail expression, the MP:GFP appeared as a single large perinuclear aggregate (Figure 4C), whereas expression of XI-K tails caused formation of several smaller aggregates (Figure 4G). The observed aggregation of the MP:GFP was not due to the higher expression levels of the myosin XI-K or XI-2 tails relative to that of myosin XI-F tail as shown by immunoblot analysis (Figure 4H).

**Figure 5:**
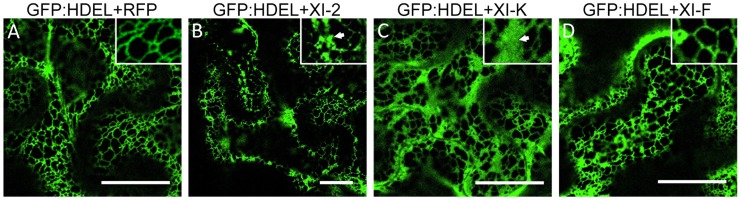
Effect of specific myosin tail expression on ER structure. A, transient expression of RFP in 16c plants at 2 dpa. ER network structure is not affected and the normal tubular ER structure is seen. B, expression of myosin XI-2 tails in 16c plants. The tubular structure of the ER is disrupted and GFP-containing aggregates are formed (arrow inside the inset). C, expression of myosin XI-K tails in 16c plants. The normal tubular structure of the ER is inhibited. Instead, large sheet-like membrane structures are seen (arrow inside the inset). D, expression of myosin XI-F tails in 16c plants. No effect on the ER structure is observed. A-D, proteins were expressed by co-agroinfiltration and observed at 1 dpa. Scale bars, 20 µm.

Since the MP also associates with the ER [9], the aberrant accumulation of MP:GFP in aggregates in the presence of myosin XI-2 and XI-K tails may be consistent with the previously described inhibition of the dynamic behavior of the ER and the formation of aberrant ER sheets upon mutational inactivation of either myosin XI-K or myosin XI-2 in Arabidopsis [30]. Indeed, co-expression of MP:RFP with myosin XI-2 tails in transgenic *N. benthamiana* plants expressing an ER marker (GFP:HDEL) revealed that the MP aggregates are associated with ER and therefore are likely ER-derived (Figure 4D). To test whether the observed effect of myosin tails on the subcellular localization pattern of MP is an indirect effect resulting from ER abnormalities, we further studied the ER structure and dynamics in GFP:HDEL plants upon expression of myosin class XI-2, XI-F, XI-K, VIII-1, VIII-2 and VIII-B tails, or of RFP as control. As expected, ER structure was not affected in plants expressing RFP (Figure 5A). However, the expression of myosin XI-2 tails led to the formation of ER aggregates (Figure 5B), whereas the expression of myosin XI-K tails led to inhibition of the normal tubular structure of the ER and the formation of sheet-like structures (Figure 5C). Normal ER network structure was observed upon expression of myosin XI-F (Figure 5D) or of the myosin VIII-1, VIII-2 or VIII-B tails (Figure S1). In addition to affecting the ER network structure, the expression of either myosin XI-2 or XI-K tails also significantly reduced the dynamic behavior of the ER membrane (Video S1 and Video S2, respectively). This inhibition of the dynamic behavior of the ER membrane was not seen upon expression of RFP (Video S3) or the tails of myosins XI-F, VIII-1, VIII-2 and VIII-B (Video S4).

**Figure 6:**
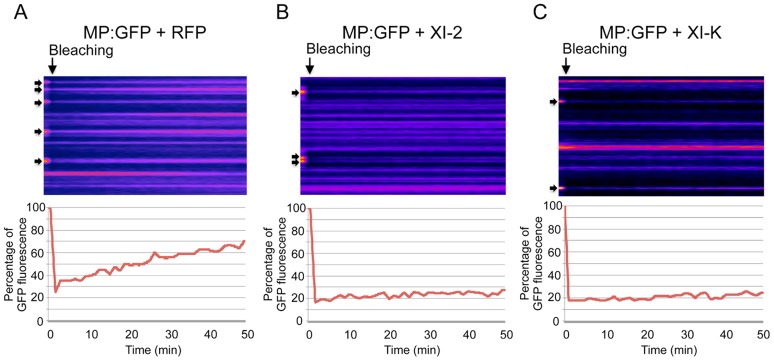
Inhibition of myosin XI-2 and XI-K reduces the efficiency by which MP is targeted to PD. A-C, Kyomographs showing examples of fluorescence recovery during the time of the FRAP experiments (50 min, from the left to the right) after bleaching of several PD (arrows) at 1 dpa. A, FRAP experiment in the presence of RFP. MP:GFP fluorescence at photobleached PD recovers to 70% during 50 minutes after bleaching (lower panel). B, FRAP experiment in the presence of myosin XI-2 tails. MP:GFP fluorescence at PD recovers only to 30% during 50 minutes (lower panel). C, FRAP experiment in the presence of myosin XI-K tails. Like in the presence of XI-2, MP:GFP fluorescence at PD recovers only to 30% during 50 minutes (lower panel). Fat arrows at the left side of the kymographs indicate the location of photobleached PD. Arrows on the top of the kymographs indicate the bleaching time point.

Although the inactivation of myosins XI-2 and XI-K had no significant effect on the PD localization of the MP, the obvious effect on the structure and dynamic behavior of the ER could affect the dynamics and efficiency of MP targeting to PD. To examine this possibility, we performed Fluorescence Recovery After Photobleaching (FRAP) assays. Upon bleaching the MP:GFP fluorescence at PD, the rate of the MP:GFP fluorescence recovery over time reflects the efficiency with which MP:GFP is delivered to PD. Figure 6 shows the results of FRAP analysis in the format of kymographs providing both the visual representation of PD fluorescence recovery over time (upper panels) and a quantitative assessment in the form of diagrams (lower panels). As expected, overexpression of RFP did not affect the recovery of MP:GFP fluorescence at PD upon bleaching: the fluorescence levels recovered to 70% of the pre-bleach level within 50 minutes (Figure 6A). In contrast, the recovery of MP:GFP fluorescence at PD was severely reduced to less than 30% upon expression of myosin XI-2 or XI-K tails (Figure 6B and C, respectively). Thus, the effects of myosin XI-2 or XI-K inhibition on the organization and dynamic behavior of the ER, as well as on the distribution of MP along the membrane correlate with a reduced efficiency of the MP in targeting to the PD and with significant inhibition of virus transport between cells.

### Class VIII myosins are involved in the proper localization and/or accumulation of the MP at PD

**Figure 7:**
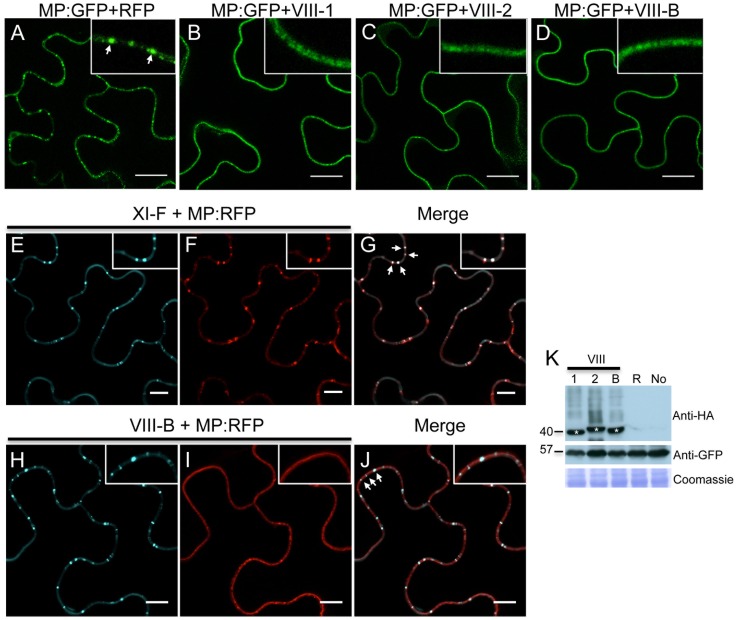
Inactivation of myosin VIII-1, VIII-2 or VIII-B disrupts the PD localization of MP. A, expression of RFP. The expression of RFP does not affect the accumulation of the co-expressed MP:GFP in PD (arrow inside the inset). B-D, expression of myosin VIII tails. The presence of either myosin VIII-1 tails (B), myosin VIII-2 tails (C), or of myosin VIII-B tails (D) interferes with the localization of MP:GFP to PD. E to G, myosin XI-F tail expression does not interfere with the co-localization of MP:RFP with aniline blue-stained callose at PD. E, aniline blue staining of callose; F, MP:RFP; G, merged images. H to J, myosin VIII-B tail expression interferes with the co-localization of MP:RFP with aniline blue-stained callose at PD. H, aniline blue staining of callose; I, MP:RFP; J, merged images. K, immunoblot analysis using HA-specific (top panel) and GFP-specific (middle panel) antibodies for the detection of myosin and MP:GFP, respectively. Bands corresponding to the class VIII (≈40 kDa) myosin tails are marked by asterisks. Coomassie blue staining (bottom panel) is shown as loading control. R, MP:GFP expressed with RFP. No, MP:GFP expressed alone. A-K, proteins were expressed by co-agroinfiltration and analyzed at 1 dpa. Scale bars, 20 µm (A-D); 10 µm (E-J).

Using the approach outline above, we also studied the role of class VIII myosins in the PD localization of MP. Strikingly, expression of the myosin VIII-1, VIII-2 or VIII-B tails drastically reduced the localization of MP at PD and caused redistribution of the MP along the cell wall (Figure 7B, C and D, respectively). This effect of myosin VIII-1, VIII-2 or VIII-B tail expression was confirmed by determining the localization of MP fused to RFP (MP:RFP) in relation to PD-localized callose stained with aniline blue. Whereas MP:RFP co-localizes with callose labeling at PD when expressed alone or when coexpressed with class myosin XI-F tail (Figure 7E-G), MP:RFP localized to the cell periphery but not adjacent to callose signal at PD when coexpressed with the tails of myosins VIII-1, VIII-2 or VIII-B (Figure 7H-J). The observed inhibitory effect of class VIII myosin tail expression on the MP targeting to and/or accumulation at PD was not caused by changes in the expression level of the MP as shown by western blot analysis (Figure 7K).

**Figure 8:**
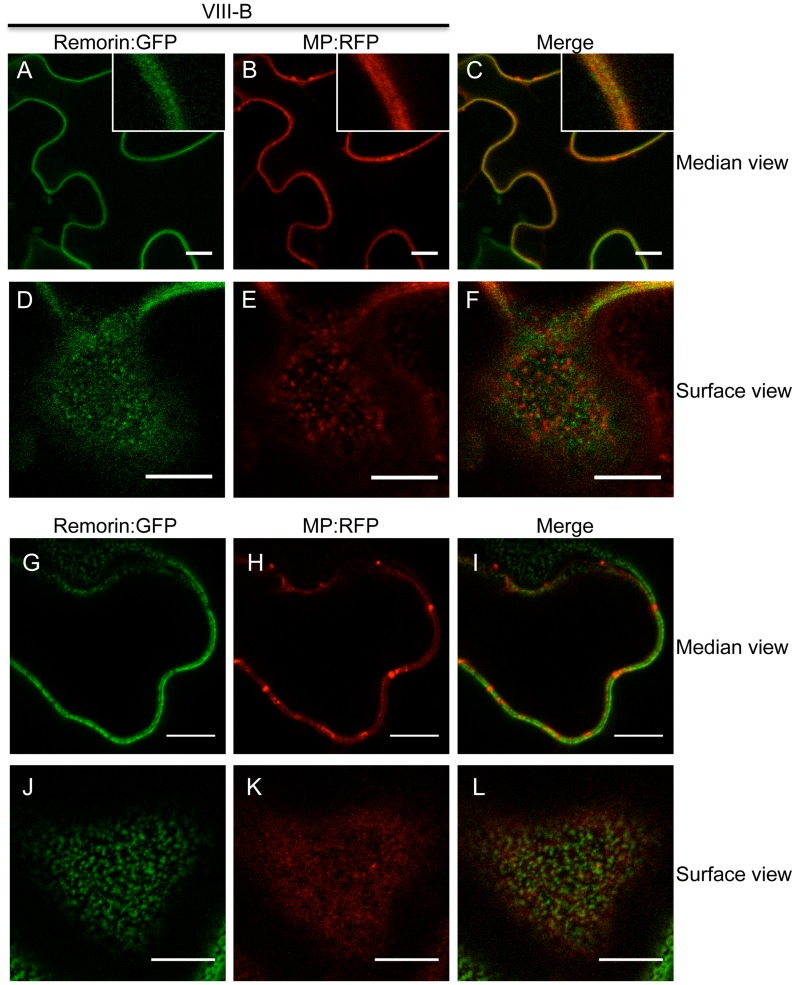
Inactivation of myosin VIII causes patchy accumulation of MP at the PM. A to C, remorin:GFP and MP:RFP co-localize at the PM in the presence of myosin VIII-B tails. A, remorin:GFP; B, MP:RFP; C, merged images. D to F, surface view on the PM. Remorin:GFP and MP:RFP localize to different patches at the PM in the presence of myosin VIII-B tails. D, remorin:GFP; E, MP:RFP; F, merged images. G to I, in the absence of myosin VIII-B tails the MP:RFP accumulates in PD. G, remorin:GFP; H, MP:RFP; I, merged images. J to L, surface view of the PM. In the absence of myosin VIII-B tails, the MP:RFP does not accumulate in patchy pattern within the PM. J, remorin:GFP; K, MP:RFP; L, merged images. Proteins were expressed by co-agroinfiltration and observed at 1 dpa. Scale bars, 10 µm.

To further investigate the TMV MP redistribution upon co-expression with myosin VIII-B tails, we in addition co-expressed remorin:GFP, which is targeted to PD and to plasma membrane (PM) lipid rafts independently of myosins XI and VIII [5], [43]. In the presence of myosin VIII-B tails, the pattern of MP:RFP distribution was similar to that of remorin:GFP, indicating that inactivation of the myosin VIII-B results in the relocalization of MP along the PM (Figure 8A-C). In PM surface view, the MP:RFP appeared in small immobile patches similar to patches of remorin:GFP (Figure 8D-E). However, there was no substantial co-localization between these proteins suggesting that the MP localized to different subdomains within or in the vicinity of the PM (Figure 8F). Co-expression MP:RFP with remorin:GFP in the absence of myosin VIII-B tails had no effect on the localization of the MP to PD (Figure 8G-I). In PM surface view the MP:RFP also did not show any significant accumulation to patches in the membrane (Figure 8J-L). Thus, the localization of MP to PM patches is an effect of myosin VIII inhibition and not caused by remorin:GFP expression.

Interestingly, myosin VIII tails were previously reported to localize to specific regions at the PM [19], which may represent lipid rafts as suggested by Tilsner et al. [44]. The pattern of MP:RFP localization to PM patches seen upon inactivation of myosin VIII may therefore indicate an interaction of MP:RFP with immobilized, potentially membrane raft-associated myosin VIII.

### Inactivation of myosin XI-2 disrupts the accumulation pattern and the subcellular localization of the 126k subunit of TMV replicase

**Figure 9:**
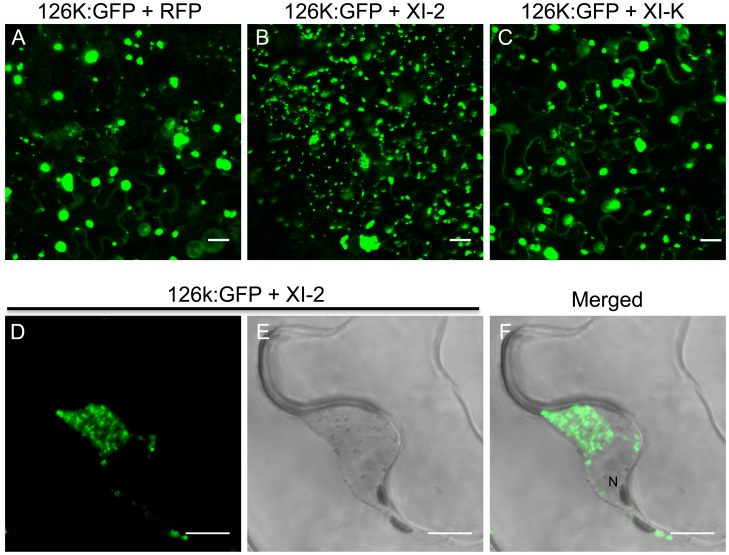
Inhibition of myosin XI-2 disrupts the normal subcellular localization of expressed 126k:GFP. A, co-expression of 126k:GFP together with RFP. The 126k:GFP accumulates in large aggregates. B, co-expression of 126k:GFP together with myosin XI-2 tails. 126k:GFP is dispersed into numerous small aggregates. C, co-expression of 126k:GFP together with myosin XI-K tails. The 126k:GFP accumulates in large aggregates similar to those seen upon expression of RFP. Similar normal aggregates are seen also upon co-expression of the 126k:GFP with either XI-F, VIII-1, VIII-2 or VIII-B tails. D-F, co-expression of the 126k:GFP together with myosin XI-2 tails. The 126k:GFP accumulates, in addition to small aggregates, in a big aggregate in the vicinity of the nucleus. A-F, proteins were expressed by co-agroinfiltration and observed at 2 dpa. Scale bars, 20 µm (A-C); 10 µm (D-F).

TMV encodes two replicase subunits, the 183 kDa (183k) and the 126 kDa (126k) proteins. The larger subunit is produced by read-through of an amber stop codon that terminates the translation of the shorter protein and harbors the RNA-dependent RNA polymerase (RdRp) domain required for replication. Although the 183k subunit is sufficient for TMV replication, the 126k subunit increases the replication rate [45] and acts as the viral suppressor of RNA interference [46], [47]. It is not clear whether the latter subunit forms part of the motile viral replication complex (VRC). Liu et al. [12] showed that transiently expressed 126k forms intracellular inclusion bodies that, similar to VRCs, align with and move along actin filaments. These findings suggest that the 126k protein may functionally contribute to the formation and trafficking of VRCs along the ER-actin network. To investigate whether the formation of 126k-containing inclusion bodies is myosin-dependent, we studied the subcellular localization of 126k and the formation of the inclusions upon inactivation of class VIII and class XI myosins. Expression of the control RFP (Figure 9A) or of myosin XI-K tails (Figure 9C), as well as of the myosin VIII-1, VIII-2, VIII-B, or XI-F tails (Figure S2) did not significantly affect the subcellular localization of 126k protein and the number or the size of inclusions. However, expression of the myosin XI-2 tails resulted in the formation of more numerous and smaller 126k inclusion bodies (Figure 9B), and also caused aggregation of 126k:GFP near the nucleus (Figure 9D-F). This perinuclear localization of 126k:GFP in the presence of myosin XI-2 tails was similar to the localization of MP seen under the same conditions (Figure 4C). Indeed, upon the expression of myosin XI-2 tails or myosin XI-K tails, the two proteins colocalize around the nucleus (Figure S3A-C) and in smaller aggregates, respectively (Figure S3D-F). This outcome is consistent with the role of myosins XI-2 and XI-K in the formation and dynamic behavior of the native ER structure (Figure 5) and suggests that the 126k protein is associated with the ER-actin network like MP. Consistently, expression of myosin XI-2 tails impaired the movement of the 126k-containing inclusion bodies along actin filaments (Figure S4). The observation of smaller 126k aggregates in the presence of myosin XI-2 tails (Figure 9B) correlates with the decrease in viral accumulation seen under the same conditions (Figure 3), and is consistent with previous reports indicating that smaller sizes of 126k-containing inclusion bodies are associated with a decrease in virus replication and with weak silencing suppression activity [12], [46]. Thus, our results support specific contributions of myosin XI-2 to the formation of larger ER-associated 126k complexes required for optimal RNA replication and the suppression of the antiviral RNA interference response.

## Discussion

Many plant and animal viruses depend on cytoskeletal elements for the entry, replication and movement within and between the cells [3], [48]–[51]. However, the specific mechanisms underlying virus interactions with the microtubules, actin filaments and associated motor proteins during phases of genome replication and intra- and intercellular movement are relatively poorly understood. Although the role of actomyosin-mediated transport of TMV to and through PD has been investigated for over a decade, no consensus has been reached, likely due to the varying experimental conditions and non-specific effects of the used chemical inhibitors [11]–[14], [39].

Recently, Harries et al. [39] employed a more advanced approach whereby the expression levels of individual myosins in TMV-infected plants were reduced by RNA interference triggered by co-infecting *Tobacco rattle virus*. This work implied a significant role of myosin XI-2 in TMV cell-to-cell movement, whereas reduced levels of the myosins XI-F, VIII-1, or VIII-2 had no apparent effect on this process. It should be noted, however, that research into myosin functions is significantly impeded by functional redundancy of these molecular motors such that even complete elimination of the individual myosins XI in corresponding gene knockouts results in little or no phenotypic effect [22], [23]. Only the simultaneous elimination of two or more myosins consistently affects cell and plant growth [24], [26], [40]. Utility of such multiple gene knockouts for virus transport research is, however, compromised by their pleomorphic developmental phenotypes that are likely to affect virus-host interactions.

It has been shown that the dominant negative inhibition of myosin activity by transient overexpression of the particular myosin tails has a stronger effect on myosin-driven transport than the elimination of the corresponding myosin gene [23], [28], [29], [52]. The *modus operandi* of the dominant negative inhibition is not entirely understood. However, we show here that the expression of myosin tails does not interfere with the accumulation of corresponding endogenous myosins (Figure S5). Although tail expression could affect the regulation of myosin activity [52], the (near) consensus in the field is that the dominant negative inhibition is due to interference with efficient cargo binding by the functionally related endogenous myosin paralogs. Mechanistically, the inhibition effect could involve cargo sequestration by headless myosin tails, as well as formation of non-functional heterodimers with full-size endogenous myosins. Here, we used the inhibition approach to mitigate the redundancy problem and to revisit the roles of myosins in TMV infection. In addition to validating a contribution of myosin XI-2, our results indicate that myosin XI-K might even be more critical for TMV cell-to-cell movement because expression of its tail reduced the virus-infected area by 75% compared to 33% in the case of myosin XI-2 tail (Figure 1B). Importantly, because expression of the myosin VIII-1, VIII-2 and VIII-B tails also resulted in two-fold reduction in local spread of TMV, we conclude that the class VIII myosins also play a significant role in virus movement from cell to cell. Consistent with these effects, inhibition of each of these class XI and class VIII myosins caused a delay or even abolished (in the case of XI-K) the long-distance transport of the virus to non-inoculated leaves and other tissues (Figure 2). Previous studies [39] did not test for effects of myosin inhibition on systemic virus movement. Because in our experiments the myosin tails were expressed only in the virus-inoculated leaves, the corresponding inhibition of systemic transport, particularly strong in the case of myosin XI-K tails, relates the efficiency by which the virus moves cell-to-cell in the inoculated leaves to the efficiency of systemic movement, as is consistent with previous observations [53]. Our results may also indicate that myosins play a distinctive role in supporting the entry of the virus into the vascular phloem.

To further understand the mechanisms by which myosins contribute to TMV infection, we examined the effects of myosin tail overexpression on the subcellular accumulation patterns of the MP and 126k proteins, the key players in regulating TMV reproduction and movement [3], [54]. It was found that the inhibition of myosins XI-2 and XI-K affected the subcellular distribution and efficiency of the PD targeting of the MP (Figure 4C-D and G). In accord with previous work [30], the change in the subcellular accumulation pattern of the MP correlated with reorganization and inhibition of the dynamic behavior of the ER (Figure 5C; Video S3 and Video S4).

We have also found that the inhibition of myosin XI-2, but not XI-K or myosins VIII, affected the level of viral RNA accumulation and the subcellular distribution pattern of the 126k replicase subunit (Figures 3 and 9). These observations are in agreement with the roles of the 126k protein as a constituent and a size regulator of the ER-associated VRCs [12], [55]. Thus, it seems plausible that myosin XI-2 contributes to the formation of larger 126k protein complexes that support more efficient amplification of the viral genome.

Intriguingly, recruitment of class XI myosins for virus transport appears to be a widespread phenomenon. The myosins XI-2 and XI-K were implicated in the PD targeting of PDLP1 [4], a host receptor required for tubule formation by the MP of GFLV, a nepovirus [5]. The *Tomato spotted wilt tospovirus*, also a tubule-forming virus, has been reported to use myosin XI-K for transporting its nucleocapsid within the cell; inhibition of this myosin delayed systemic infection by this virus [37]. Viruses so far reported to require myosins VIII for the PD targeting of their MPs include *Beet yellows closterovirus* [34], *Rice stripe tenuivirus* [35] and *Rice black-streaked dwarf fijivirus* [38]. Each of these otherwise dissimilar viruses employs a cell-to-cell movement mechanism that does not involve PD tubule formation. As we show here, this pattern is shared by TMV, also a non-tubule-forming virus.

Indeed, the inactivation of myosins VIII-1, VIII-2 or VIII-B disrupted the PD localization of the TMV MP and caused an unusual accumulation of MP at the PM under these conditions (Figure 7). However, unlike inhibition of class XI myosins, the inhibition of class VIII myosins had no effect on the ER organization and did not cause MP retention at the ER. Therefore, a significant reduction in virus transport between cells upon myosin VIII inhibition (Figure 1B) is likely associated with a later event that depends on PD localization of the MP. Accordingly, we propose that the MP traffics along the ER with the aid of class XI myosins and that class VIII myosins are involved in a transport of MP between ER and the PM-lined PD, a pathway that normally prevents the accumulation of MP along the PM. This is in line with a concept of localized endocytotic cycling pathways that mediate and maintain protein shuttling between the ER and specific PM domains, including the PM domain at PD [10]. Additional support for this concept is provided by the reported localization of class VIII myosins at PD [17]–[19], a proposed role of class VIII myosins in endocytosis [19], [20], and by MP interaction with the synaptotagmin-like protein SYTA [56], a regulator of endocytosis clustered in lipid raft-like PM domains [57]. Further elaboration of this concept includes cortical microtubule-associated ER sites (C-MERs) that may provide anchorage platforms for protein recruitment and subsequent actomyosin-dependent transport to the PM and PD hijacked by viruses for their intercellular movement [10].

**Figure 10:**
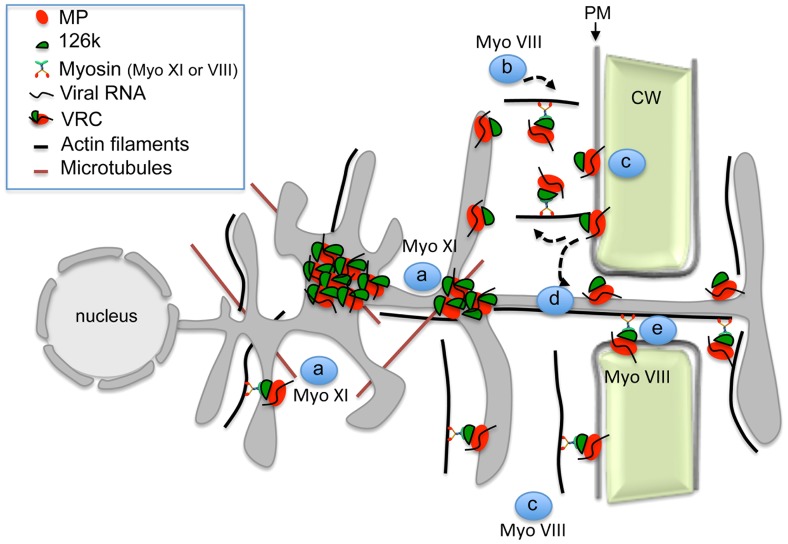
Model illustrating the role of the actomyosin system in the intra- and intercellular movement of TMV. Myosins XI-2 and XI-K provide motility to the ER and facilitate the concentration of VRCs at cortical ER sites in the vicinity of the PM (a). VRCs are concentrated and stabilized by the 126k through myosin XI-2 function to enhance viral replication and silencing suppression (a). Class VIII myosins are involved in the targeting of the MP/VRCs from the cortical ER sites to the PM and subsequently to PD. This process may involve endocytic recycling (b), diffusion of the MP/VRC along the PM (c), stabilization of MP/VRC at PD (d) and active transport through the channel into the adjacent cell (e).

Together with previous studies, our work supports a more detailed mechanistic model of the interplay between TMV and the actomyosin system (Figure 10). This model proposes that the myosins XI-2 and XI-K mobilize the dynamic ER-actin network and facilitate concentration of VRCs at C-MERs, specific sites of the cortical ER adjacent to the PM. The 126k protein stabilizes the VRC cluster at these sites with support of myosin XI-2 and thus enhances viral amplification. An endocytic recyling pathway that links the cortical ER-associated VRC clusters to the PM domain at or near PD, maintains the localization of MP within this domain and thus facilitates directed macromolecular transport from the ER to PD and into the adjacent cell. Myosin VIII is a principal driver of these final steps of viral transport between cells that activates endosomal trafficking and also prevents the distribution of the MP along the PM. In conclusion, our data show that individual myosins VIII and XI provide distinct functional contributions to specific steps in TMV intra- and intercellular movement.

## Materials and Methods

### Plant material and virus inoculation

All experiments were performed using 5-week-old *Nicotiana benthamiana* wild type plants or 16c transgenic plants stably expressing 35S:GFP-HDEL [58]. The plants were grown in growth chambers under 16/8h light/dark cycles, 24/20°C day/night temperatures and approximately 70% humidity. Agro-infiltrated and/or virus-infected leaves were maintained at the same conditions. Leaves were rub-inoculated with 150 ng of purified virions (TMV-GFP-JL24) or with 3 µl of in-vitro transcription reaction mix (RiboMAX, Promega) per leaf (TMV-GFP).

### Transient protein expression

The binary vectors designed to express HA-epitope-tagged *N. benthamiana* myosin tails VIII-1, VIII-2, VIII-B, XI-K, XI-F, and XI-2 were described earlier [34]. The constructs used for transient expression of MP:GFP, MP:RFP and remorin:GFP are described elsewhere [43], [59], [60]. The construct to express 126k:GFP was generated by GATEWAY cloning according to the manufacturer's instructions (Invitrogen). The 126k open reading frame was PCR-amplified from TMV-U1 (NC_001367) and recombined into pDONR/Zeo (Invitrogen). After DNA sequence confirmation, the entry clone was used for recombination with the destination vector pB7FWG2 [61]. The presence of the anticipated insert was verified by DNA sequencing. The binary expression vectors were transformed into *Agrobacterium tumefaciens* (strain LBA4404) and used for agroinfiltration with agrobacteria grown to a final optical density (OD 600 nm) of 0.2 for expression of MP:GFP, MP:RFP and remorin:GFP and of 0.4 for expression of myosin tails [5]. Leaf samples were processed for imaging or immunoblot analysis at 24 or 48 hours post infiltration.

### Immunoblot analysis

Total protein extracts were obtained by grinding *N. benthamiana* leaf disks in Laemmli buffer, separated by 12.5% SDS-PAGE and blotted onto polyvinylidene difluoride membrane (Millipore). Immunoblot labeling was performed with anti-HA-peroxidase antibodies (Sigma-Aldrich) or with anti-GFP antibody (Invitrogen) and peroxidase-labeled secondary antibodies (Invitrogen) for luminescence detection (Roche).

### Measurement of fluorescent infection sites and statistical analysis

Photographs of TMV-GFP fluorescent infection sites in *N. benthamiana* leaves transiently expressing RFP in one half leaf and myosin tails in the other half were taken under UV light at 4 dpi, and the level of fluorescence as well as the areas of fluorescent infection sites in each half leaf were measured using ImageJ (1.47s) software. All imaging was conducted under identical illumination and exposure conditions to allow comparisons. Mean values of areas of at least 23 infection sites per treatment and representing three independent experiments were calculated. The mean values of infection site areas in half leaves expressing myosin tails were compared to mean values of infection site areas in half leaves expressing free RFP. The same infection site areas were used to measure and compare the GFP fluorescence intensity between half leaves expressing myosin tails and free RFP. To compare virus replication, the fluorescence intensity of the infection sites in leaf areas expressing myosin tails was normalized to the fluorescence intensity of the infection sites in leaf areas expressing free RFP. Statistical analyses were performed with R software. In addition, statistical ANOVA or Student's t-tests where applied where appropriate.

### Virus quantification

Virus quantification was carried out according to Niehl et al. [62]. Single infection sites of TMV-GFP formed in the presence of expressed myosin tails or RFP as control were excised at 4 dpi and total RNA was extracted using TR-Reagent (SIGMA) according to the manufacturer's instructions. RNA of TMV-GFP was quantified by qRT-PCR using 50 ng of total RNA and specific primers and probe [63]. qRT-PCR with known concentrations of TMV RNA in the solution was used to establish a standard curve from which the number of TMV-GFP RNA copies per ng of total RNA was determined. Statistical significance between the different treatments was determined by *t-test*.

### Aniline blue staining

To visualize PD-located callose, leaf disks were vacuum infiltrated with aniline blue solution (0.1% aniline blue in 67 mM phosphate buffer pH 8). Leaf disks were incubated in the dark at room temperature for 15 minutes before imaging using a Zeiss LSM780 laser scanning confocal microscope.

### Confocal laser scanning microscopy and image processing

Excised leaf disks were mounted on a microscope slide and vacuum infiltrated with water and epidermal cells imaged using a Zeiss LSM780 laser scanning confocal microscope under multitrack mode. Excitation/emission wavelengths were 488 nm/505 to 545 nm for GFP and 543 nm/585 to 615 nm for RFP. Confocal images were processed using ImageJ (1.47s) software.

### FRAP of PD

FRAP was performed with a Zeiss LSM780 laser scanning confocal microscope. PD labeled with MP:GFP in leaves expressing myosin tails or RFP as control were imaged with a 488 nm laser at 20% intensity before bleaching. Selected PD were then bleached using the 488 nm laser at 100% intensity. Subsequently, the fluorescence of the photobleached PD was recorded each minute during 50 min at 20% 488 nm laser intensity. Images were analyzed with ImageJ (1.47) software. To obtain recovery profiles corrected for potential X, Y and Z-axis drift during the acquisition time, serial Z sections over a typical distance of 6 µm were acquired at each time point. Maximum intensity projections were prepared for every time point across the time series. The projected images were then further registered with the StackReg ImageJ plugin [64] using rigid body transformation. Kymographs images displaying fluorescent intensities over time along the cell walls were obtained by manually adjusting a spline line selection along the cell walls and by reslicing the XYT volumes accordingly. Time versus fluorescence intensity data were finally obtained by plotting profiles along horizontal lines placed at the positions of the photobleached areas.

### Analysis of myosin expression by semiquantitative RT-PCR

Leaf disks of agroinfiltrated leaf areas expressing myosin XI-2 or VIII-2 tails at 2 dpa were used to extract total RNA with TR-Reagent (SIGMA) according to the manufacturer's instructions. Leaf disks of agroinfiltrated leaf areas expressing RFP were used as control. After DNase treatment, the total RNA was used for reverse transcription using random hexamer primers. The subsequent PCR reactions were carried out with specific primers that were designed by Primer3 software (http://biotools.umassmed.edu/bioapps/primer3_www.cgi) and specific for the myosin motor region, which is not present in the expressed myosin tails. (Myosin XI-2: left primer: GACGAAGCTGGCTTATTTGC/right primer: GACAGCAGCTCCTGAAATCC; Myosin VIII-2: left primer: CAAGCAATTGGCAGTGAAAA/right primer: CCCGATGGATTTCTTCTCAA). Aliquots of reverse transcription products from myosin XI-2 or VIII-B tails- or their RFP control-treated samples were used to amplify the corresponding endogenous myosins and GAPDH as housekeeping gene. Aliquots taken after 30 and 40 PCR cycles were loaded in a 2% agarose gel and stained with ethidium bromide.

## Supporting Information

Figure S1 **Expression of myosin VIII tails does not affect ER network structure.** A-C, ER network structure in 16c plants transiently expressing myosin VIII-1 tails (A), myosin VIII-2 tails (B), or VIII-B tails (C) at 2 dpa. Scale bars, 20 µm.
(TIF)

Figure S2 **Expression of myosin XI-F, VIII-1, VIII-2 or VIII-B tails does not disrupt the normal subcellular localization of 126k:GFP.** A-D, Localization pattern of 126k:GFP in *N. benthamiana* epidermal cells in the presence of myosin XI-F tails (A), myosin VIII-1 tails (B), myosin VIII-2 tails (C), or myosin VIII-B tails (D). Proteins were expressed by co-agroinfiltration and observed at 2 dpa. Scale bars, 20 µm.
(TIF)

Figure S3 **The 126k:GFP and the MP:RFP colocalize near the nucleus upon expression of myosins XI-2 and XI-K tails.** A-C, 126k:GFP (A) and MP:RFP (B) colocalize in the vicinity of the nucleus upon expression of myosin XI-2 tails (C). D-F, 126k:GFP (D) colocalizes with the MP:RFP (E) in some aggregates (F, arrows). Proteins were expressed by co-agroinfiltration and observed at 1 dpa. Scale bar: 10 µm.
(TIF)

Figure S4 **Time-lapse video frames showing expression of the 126k:GFP and the actin marker ABD:RFP in the absence (A-D) and presence of myosin XI-2 tails (E-H).** In the absence of myosin XI-2 tails the 126k:GFP-containing aggregates move along actin (arrows, A-D). Expression of myosin XI-2 tails impaired the movement of the 126k:GFP-containing aggregates along actin (Arrows, E-H). Proteins were expressed by co-agroinfiltration and observed at 2 dpa. Scale bar, 10 µm.
(TIF)

Figure S5 **Expression of myosin tails does not trigger silencing of the corresponding endogenous myosins.** A, immunoblot analysis of leaf sections using HA- antibodies revealed the expression of the HA-tagged myosin tails at 2 dpa. Immunostained bands corresponding to class XI (≈100 kDa) and class VIII (≈40 kDa) myosin tails are marked by asterisks. Coomassie blue staining (bottom panel) is shown as loading control. B, semiquantitative RT-PCR analysis of endogenous myosin mRNA. Expression of myosin XI-2 and VIII-2 tails, which are representatives of their corresponding myosin class, did not trigger silencing of the endogenous corresponding myosins in the same leaf areas also used for immunoblot analysis (A). The expression of endogenous myosins remains similar as in the control, RFP-expressing tissue.
(TIF)

Video S1 **Live video microscopy of reduced ER dynamics upon transient expression of myosin XI-2 tails.**
(AVI)

Video S2 **Live video microscopy showing drastic reduction in ER dynamics upon transient expression of myosin XI-K tails.**
(AVI)

Video S3 **Live video microscopy showing the absence of changes in ER dynamics upon transient expression of RFP.**
(AVI)

Video S4 **Representative live video microscopy showing the absence of changes in ER dynamics upon transient expression of myosin XI-F tails.** A similar absence of effects on the structure and dynamic behavior of the ER was observed upon transient expression of myosins VIII-1, VIII-2 or VIII-B tails.
(AVI)
