# Supplementary figures and images for: Myosins VIII and XI Play Distinct Roles in Reproduction and Transport of Tobacco Mosaic Virus
Source: PLoS Pathog. 2014 Oct 16;10(10):e1004448. doi: 10.1371/journal.ppat.1004448 (PMC4199776; doi:10.1371/journal.ppat.1004448)

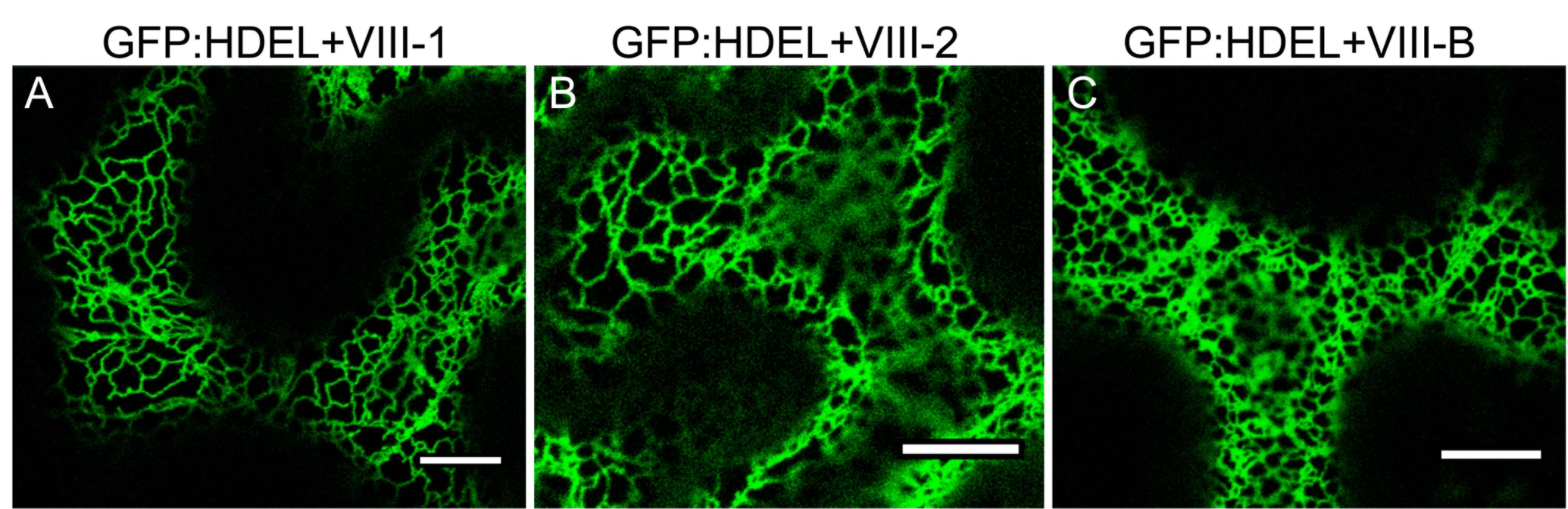

Supplement: Figure S1 — Expression of myosin VIII tails does not affect ER network structure. A-C, ER network structure in 16c plants transiently expressing myosin VIII-1 tails (A), myosin VIII-2 tails (B), or VIII-B tails (C) at 2 dpa. Scale bars, 20 µm. (TIF) [file ppat.1004448.s001.tif]

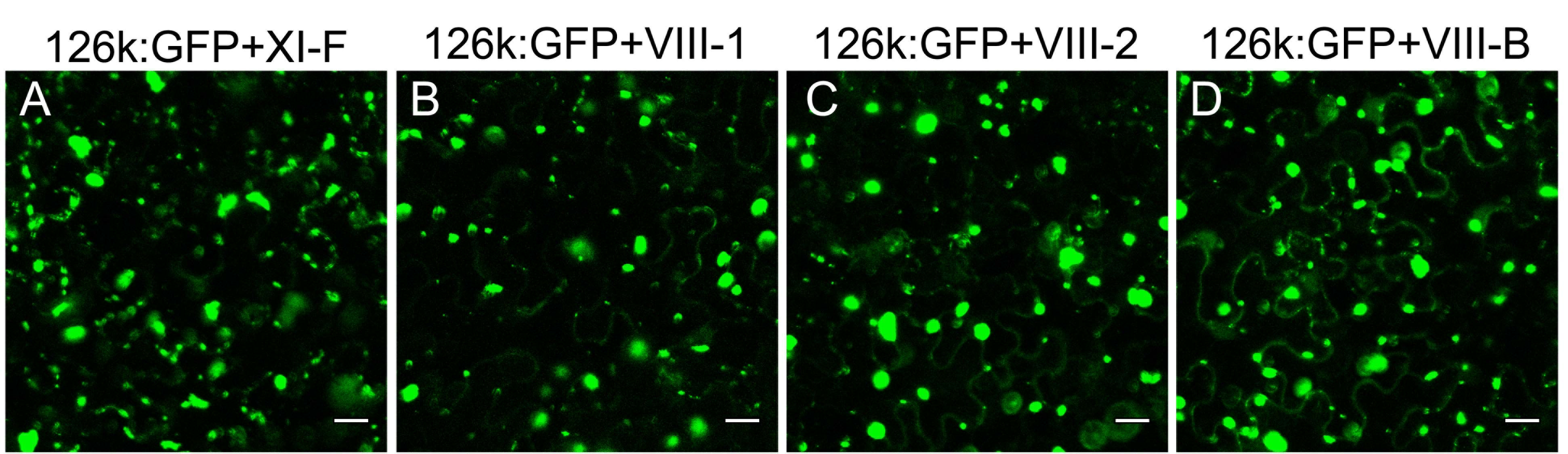

Supplement: Figure S2 — Expression of myosin XI-F, VIII-1, VIII-2 or VIII-B tails does not disrupt the normal subcellular localization of 126k:GFP. A-D, Localization pattern of 126k:GFP in N. benthamiana epidermal cells in the presence of myosin XI-F tails (A), myosin VIII-1 tails (B), myosin VIII-2 tails (C), or myosin VIII-B tails (D). Proteins were expressed by co-agroinfiltration and observed at 2 dpa. Scale bars, 20 µm. (TIF) [file ppat.1004448.s002.tif]

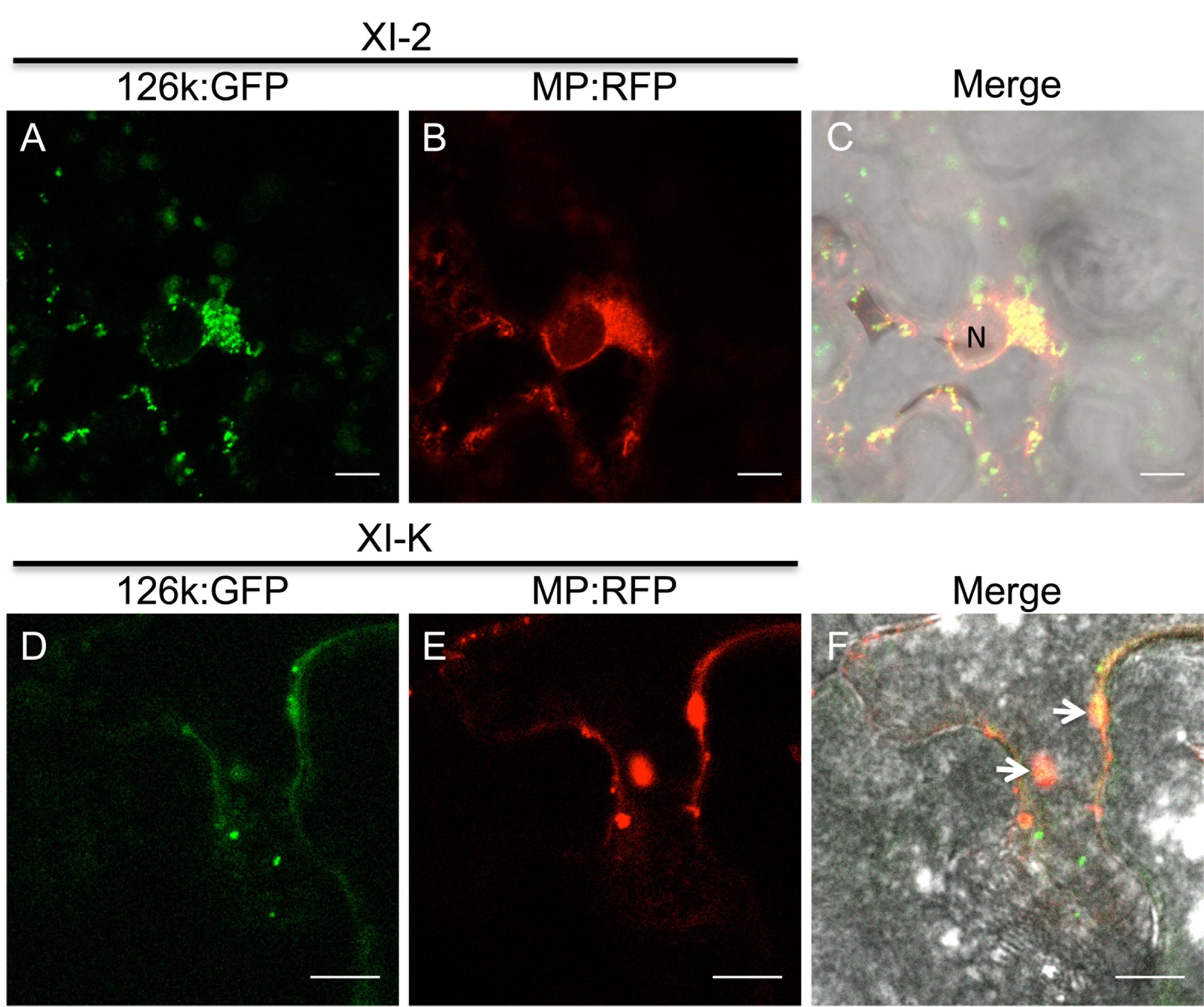

Supplement: Figure S3 — The 126k:GFP and the MP:RFP colocalize near the nucleus upon expression of myosins XI-2 and XI-K tails. A-C, 126k:GFP (A) and MP:RFP (B) colocalize in the vicinity of the nucleus upon expression of myosin XI-2 tails (C). D-F, 126k:GFP (D) colocalizes with the MP:RFP (E) in some aggregates (F, arrows). Proteins were expressed by co-agroinfiltration and observed at 1 dpa. Scale bar: 10 µm. (TIF) [file ppat.1004448.s003.tif]

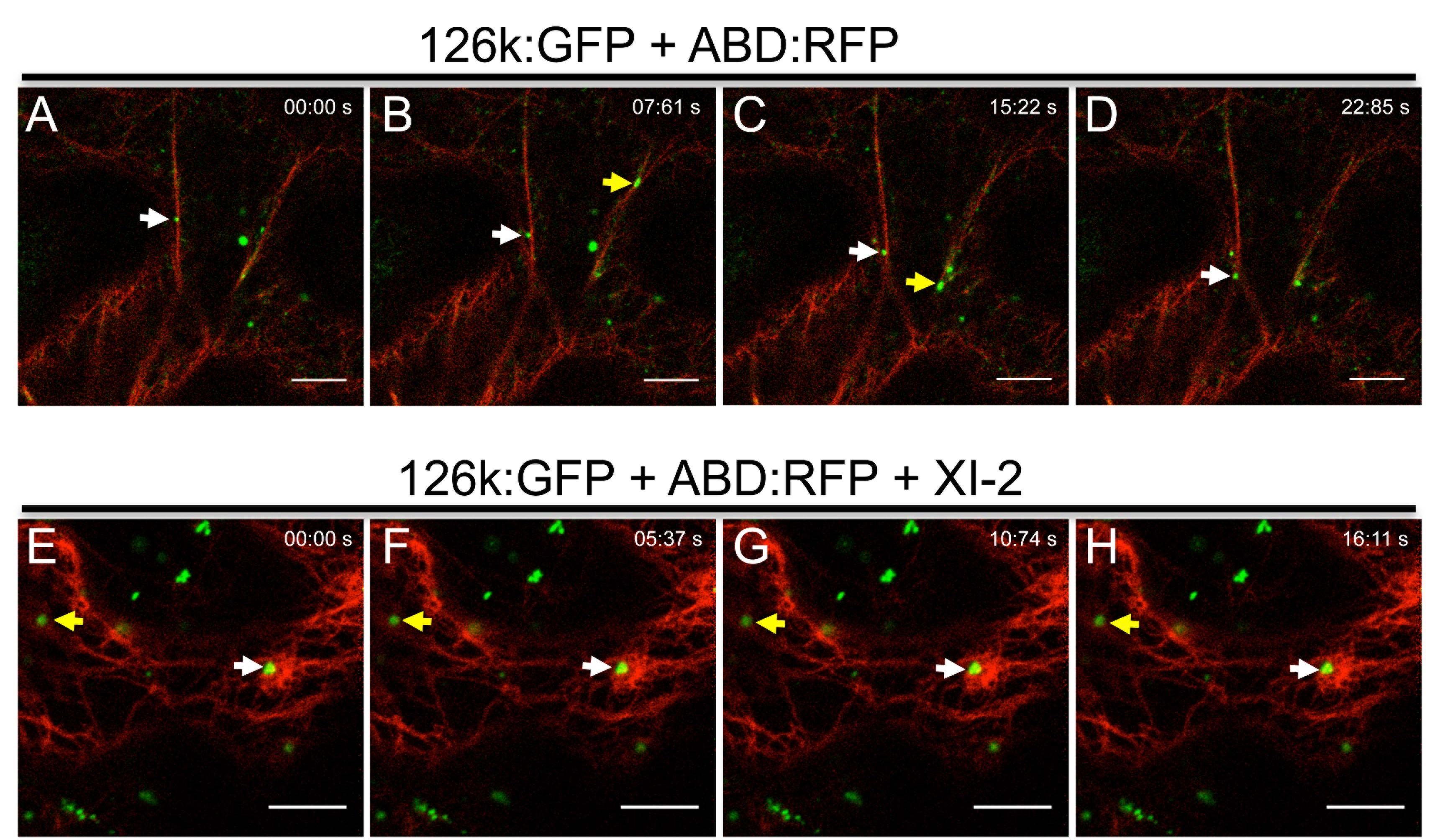

Supplement: Figure S4 — Time-lapse video frames showing expression of the 126k:GFP and the actin marker ABD:RFP in the absence (A-D) and presence of myosin XI-2 tails (E-H). In the absence of myosin XI-2 tails the 126k:GFP-containing aggregates move along actin (arrows, A-D). Expression of myosin XI-2 tails impaired the movement of the 126k:GFP-containing aggregates along actin (Arrows, E-H). Proteins were expressed by co-agroinfiltration and observed at 2 dpa. Scale bar, 10 µm. (TIF) [file ppat.1004448.s004.tif]

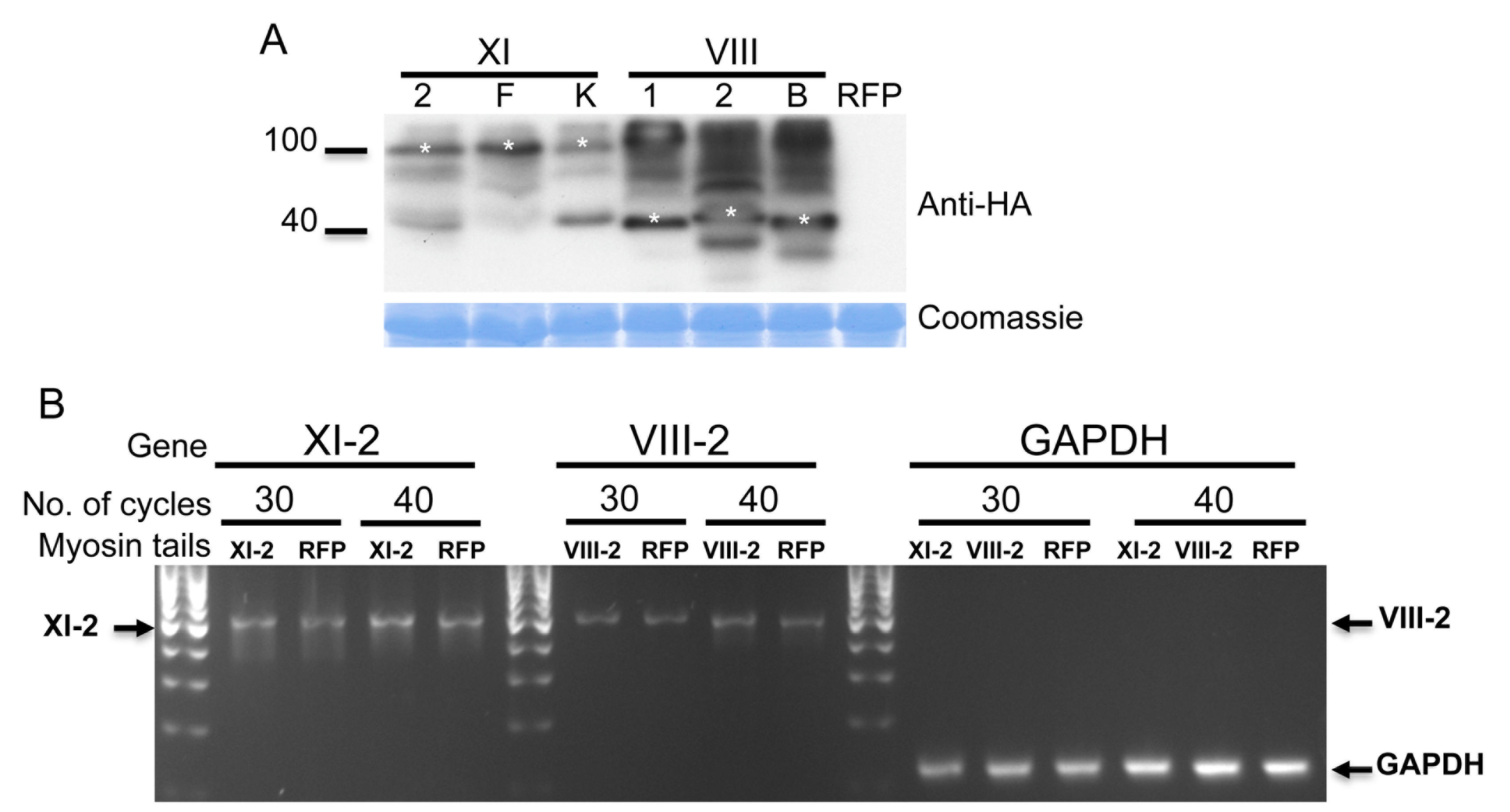

Supplement: Figure S5 — Expression of myosin tails does not trigger silencing of the corresponding endogenous myosins. A, immunoblot analysis of leaf sections using HA- antibodies revealed the expression of the HA-tagged myosin tails at 2 dpa. Immunostained bands corresponding to class XI (≈100 kDa) and class VIII (≈40 kDa) myosin tails are marked by asterisks. Coomassie blue staining (bottom panel) is shown as loading control. B, semiquantitative RT-PCR analysis of endogenous myosin mRNA. Expression of myosin XI-2 and VIII-2 tails, which are representatives of their corresponding myosin class, did not trigger silencing of the endogenous corresponding myosins in the same leaf areas also used for immunoblot analysis (A). The expression of endogenous myosins remains similar as in the control, RFP-expressing tissue. (TIF) [file ppat.1004448.s005.tif]
